# Quality of life in the elderly: A community study 

**DOI:** 10.22088/cjim.14.3.543

**Published:** 2023

**Authors:** Emitis Jazayeri, Shahla Kazemipour, Seyed Reza Hosseini, Majid Radfar

**Affiliations:** 1 Department of Social Sciences, Faculty of Humanities, Science and Research Branch, Islamic Azad University, Tehran, Iran; 2 Faculty of Social Sciences, University of Tehran, Tehran, Iran; 3 Social Determinants of Health Research Center, Health Research Institute, Babol University of Medical Sciences, Babol, Iran; 4 Department of Social Sciences, Rudehen Branch, Islamic Azad University, Rudehen, Iran

**Keywords:** Health indicators, Physical health, Mental health, Social health

## Abstract

**Background:**

Standard information about the physical, mental, and social status of older people is needed to promote their health. The aim of this study was to determine the physical, mental and social health status of older people in Mazandaran, taking into account the specific climatic conditions and public culture, and to adapt it to the indicators of the "Aging and Health Program" of the World Health Organization (WHO) to localize and better use this index.

**Methods:**

In this cross-sectional study, 390 elderly aged ≥65 years in Mazandaran were selected by the quota method. Their physical, mental, and social health status and demographic information were collected using a questionnaire (a 36-item short-form health survey (SF-36)) and face-to-face interviews. Physical and mental health status was classified into five levels based on the score obtained. The data were analyzed using SPSS 26.

**Results:**

The average age of the elderly was 71.48 years. In terms of physical performance, 40.7% of the elderly were below and 30.8% above the average of international standards. In terms of mental health, 18.9 and 41.5% of the elderly were below and above the average, respectively, and 17 and 51.8% of the elderly had low and high social performance, respectively. The Pearson correlation test showed a significant relationship between physical health (0.176), mental health (0.16), and social health (0.178) and quality of life at the 0.01 level.

**Conclusion:**

The physical, mental and social health status of the elderly in Mazandaran is far from the indicators of health in old age established by WHO, and their condition is unfavorable (at least in terms of physical and mental health).

One of the most important demographic issues that has attracted much attention is the aging of the population. Economic and social development, medical advances, declining fertility rates, and increasing life expectancy have led to significant changes in the structure of the world's population in recent years. During this period, the number of elderly people has increased significantly, especially in developing countries.

Since the phenomenon of aging causes significant changes in all aspects of human life, including a wide range of age structures, norms, values and the establishment of social organizations, it is very important to address the challenges of this phenomenon and take appropriate measures to improve the physical, psychological and social status of the elderly. Moreover, today it is not just about staying alive, it is also about quality of life (QOL) and how you live. Supporting older people should not be done only with the aim of increasing their life expectancy. Nowadays, dynamic aging is the goal, which means that as the elderly population increases, their QOL should also be considered (1).

Due to the increase of the elderly population in the country, it is necessary to pay attention to the well-being of the elderly in physical, mental and social aspects as the needs of the elderly population. To promote the health of the elderly and prevent and reduce their diseases, it is necessary to first determine the condition of the elderly.

One of the indicators that reflects the condition of the elderly well is the QOL index. According to WHO, QOL is "the perception of one's own life situation in the context of cultural conditions, value system, and in relation to one's goals, norms, and interests." Age, gender, health status, and cultural factors are important factors that influence people's perceptions of QOL. Although the QOL of older people declines as they age, other factors also contribute to this decline. Most older people suffer from chronic diseases and receive constant treatment, which can affect their QOL. Therefore, knowledge about the QOL of the elderly is essential for optimal care and supportive interventions (2).

The 2015 United Nations projections have shown that although Iran's population growth will gradually decline over the 2011-2050 period, the overall population will increase by the 21st century. If the average United Nations scenario is achieved (i.e., a total fertility rate of 1.62 and its relative stability during 2015-2020, and an increase in life expectancy to 79 years by 2050), the proportion of elderly people in Iran will reach 14.4% by 2030 and 31.2% by 2050. In 2050, the proportion of elderly people will be 21.5% in the world and 24% in Asia, and the proportion of elderly people in Iran will be higher than the average of elderly people in the world and Asia, so that every third person will be an elderly person (3). 

In the studies conducted so far, the scope and range of variables related to the elderly are very limited, and in most cases, they refer to the health information of the elderly, while less attention has been paid to the social dimensions of their lives, (4) although the characteristics and social conditions of the lives of the elderly are no less important than their physical health. In recent years, research on the relationship between the social support network and health has gained importance (5-6). 

Studies have shown that the characteristics of a person's social support network and its function can influence the consequences for physical, cognitive, and mental health, overall social functioning, and participation in older people (7). Researchers also discuss whether the quantity (characteristics) and quality (social support) of social support networks may differentially affect health (8). However, the characteristics of the social support network in different societies are highly influenced by their cultural background (9,10). Studies have indicated that more support from friends and spouses is related to QOL in older Canadians, whereas in Latin America, family support is associated with better emotional and physical health (11). The results of Li et al. revealed that support from friends was more helpful than support from family in promoting physical health (12).

Considering the above, researching the effective factors for healthy aging should be one of the most important goals of national macro policy. The elderly population of Mazandaran was considered as the target population in the current study. Mazandaran has an area equivalent to 1.46% of Iran's land area, hosts more than 4.1% of the country's population, and is one of Iran's most populous regions in terms of population density in 2016, after Tehran, Alborz, and Guilan provinces (population density: 127.7 persons per square kilometer). 

The growth of the elderly population in Mazandaran is becoming a major challenge for healthcare providers, family members, and the community in which the elderly live. According to the findings of Kusheshi (2013) in a study titled "Iranian aging population" (demographic and socio-economic characteristics and challenges ahead), the three provinces of Guilan, Mazandaran, and Tehran have the highest proportion of population aged ≥65 years compared to other provinces in the south, southeast, and southwest of Iran such as Sistan and Baluchestan, Ilam, Kohgiluyeh and Boyer-Ahmad, Hormozgan and Bushehr, Khuzestan, and provinces in the northeast and west such as West Azerbaijan.

According to the 2016 census, the sex ratio of the elderly in this province is 96-97 (with a range close to the average of the whole country), which is in line with the pattern of gender composition of the feminization of the aging population in Iran and most countries. In addition to the gender composition, the role and importance of the literacy rate of the elderly can also be highlighted. The literacy rate of the elderly does not have uniform significant differences in social inequality are observed among the provinces of the country. The aim of this study was to identify the physical, mental and social dimensions of health of the elderly and its relationship with the QOL of the elderly. 

## Methods

The present study which was approved by the Research Council of Islamic Azad University Science and Research and received the ethical code of Babol University of Medical Sciences (IR.MUBABOL.HRI.REC.1400.129), was conducted from January 2017 to December 2016 on 390 elderly aged ≥65 years selected from 248269 inhabitants of Mazandaran (based on the last population and housing census (2016)) using the quota method. After making the list of cities in Mazandaran, according to the statistics of the elderly in the urban areas of the province (Table 1), based on gender and the proportion of each region in the sample size, the questionnaires were completed through face-to-face interview. Inclusion criteria for the elderly in this study included age ≥65 years, willingness to participate in the study, resident of Mazandaran, and ability to answer the questions, while exclusion criteria were unwillingness to cooperate further. Participants were informed that the results of the study would be kept confidential. Public places in the city, including mosques, parks, weekly markets, and clubs, were used to facilitate reaching the elderly. The sample size (n) was 384 based on Cochran's formula at an error level of 0.05%.

$n=\frac{248269*1.96^{2}\mathrm{*0.5*0.5}}{248269*0.05^{2}-1.96^{2}\mathrm{*0.5*0.5}}=384$

On the other hand, assuming that the elderly might not answer the questionnaires correctly in some cases due to their age, more questionnaires were distributed (about 450), and finally, 390 correct and accurate questionnaires were evaluated. To evaluate the physical, mental and social health status, one of the questionnaires used was the researcher-made questionnaire (demographic information and personal and general characteristics of the respondents) and the other was the 36-item short-form health survey (SF-36) was used to assess the physical, mental and social health status. The SF -36 questionnaire has been shown to be suitable for clinical research and practice, general population surveys, and health policy evaluation. It was developed in 1992 by Ware and Sherbourne in the United States, and its validity and reliability were studied in different groups (13). This questionnaire was adapted to the social and cultural conditions of Iran by Ali Montazeri in 2005 (14). The concepts measured by this questionnaire are not specific to a particular age, group, or disease. The purpose of this questionnaire was to assess health status in terms of physical, mental, and social status, which was determined by combining the scores of the eight health domains.The questionnaire includes 36 questions that measure 8 different health domains, so each domain consists of 2-10 questions. The questionnaire also includes a question in which the person evaluates his or her health status in the last month. To assess the respondent's physical health, there are 21 questions in four subscales, including role limitations and physical health status, general health status, physical functioning, and bodily pain. To measure mental and social health, there are 14 questions in four subscales including energy and vitality, emotional health, role limitations due to emotional problems, and social functioning. Each question is used only to calculate the score of a scale. For some questions, the scores are coded again so that all scales are in the same direction. The scores for each domain are converted into a score between 0 and 100, with higher scores indicating a higher level of QOL (0= the worst and 100= the best). At the end, the average scores of two general subscales for physical and mental health are obtained and calculated. In addition to the SF-36 questionnaire, some questions were included in the questionnaire by the researchers to evaluate effective social factors and their health dimensions. Thirty questionnaires were evaluated as a pretest, and the final corrections were made to the questionnaire based on the experiences and comments of supervisors and advisors.

**Table 1:** The number of population aged 65 years and older (2016) and the size of the selected sample in the cities of Mazandaran province

**City**	**Number of population aged ≥65**	**Sample size**	**City**	**Number of population aged ≥65**	**Sample size**
Seemorgh	1688	3	Amol	27694	43
Abbasabad	3526	5	Babol	42416	66
Fereydunkenar	4015	6	Babolsar	9227	14
Qaemshahr	23718	37	Behshahr	12239	19
Kelardasht	2183	3	Tonekabon	15546	24
Galugah	3281	5	Ramsar	7364	11
Mahmudabad	6704	10	Juybar	5712	9
*Miandorod*	4259	7	Chalus	8808	14
Neka	7458	12	Sari	35157	54
Nur	9590	15	Savadkuh	3999	6
Nowshahr	10835	17	Savadkuh-e-Shomali	2850	4
Mazandaran province	Number of population aged ≥65			248269	
	Sample size			384	

Finally, the questionnaire on the indicators of health in old age in the dimensions of physical health (Cronbach's alpha coefficient=0.76), emotional health (Cronbach's alpha coefficient=0.96), mental health (Cronbach's alpha coefficient=0.82), social performance (Cronbach's alpha coefficient=0.71), well-being (Cronbach's alpha coefficient=0.93), presence of a sense of purpose in life (Cronbach's alpha coefficient=0.72), QOL (Cronbach's alpha coefficient=0.84), life satisfaction (Cronbach's alpha coefficient=0.75), fulfillment of needs (Cronbach's alpha coefficient=0.78) and individual perceptions (Cronbach's alpha coefficient=0.89) was prepared. The collected data were analyzed using SPSS 26 and descriptive tests and inferential statistics (Pearson correlation coefficient, independent t-test, one-way analysis of variance) at a significance level of <0.5.

## Results

**Figure 1:**
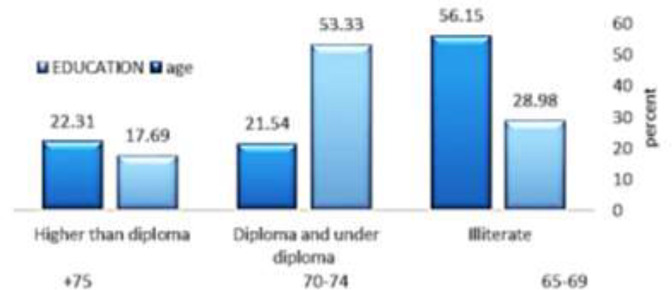
Demographic characteristics of the elderly in Mazandaran by age group

Demographic characteristics of the respondents the number of elderly men and women was equal (men=women=195). The mean age of the participants was 71.48±6.363 years with an age range of 96-65 years, and the mean ages of men and women were 71.36±6.08 and 71.59±6.647 years, respectively. The figure below shows the demographic characteristics of the statistical population. According to the results, most of the elderly studied were between 65 and 69 years old and with high school diploma and below-high school diploma (Figure 1).

**Descriptive results**

**Physical health domain of the elderly: **To assess respondents' physical health, four subscales, including role limitations and physical health status, general health status, physical functioning, and bodily pain, were measured. The overall physical health score was then derived by combining the scores from these four domains. Table 2 illustrates the percentage distribution of scores obtained by respondents by physical health domain. The figures in this table indicate that about one-third of the elderly respondents are average in physical health, less than one-third are below average in physical health, and the overall health of 30% of the elderly respondents is above average. 

**Mental health domain of the elderly: **To assess mental health, 3 subscales were used: energy and vitality, emotional health, and role limitation due to emotional problems. Table 3 shows the percentage distribution of scores obtained by respondents by mental health domain.

In the present study, about 28.7% of the elderly rarely felt vital and alive, about 37% of them had impaired emotional health, and about 37.8% of the elderly respondents were severely or very severely restricted in their roles due to emotional problems. According to the index of general mental health, about one-third of the elderly had high, moderate, or low levels of mental health. 

**Table 2:** Percentage distribution of scores obtained by respondents by physical health domain

	**Very low**	**Low**	**Moderate**	**High**	**Very high**	**Total**
**General health**	2.1	21.5	64.1	11.8	0.5	100
**Physical functioning**	17.4	23.3	28.5	21.3	9.5	100
**Role limitations and physical health status**	6.4	24.9	38.2	25.9	4.6	100
**Bodily pain**	Very little	Little	Relatively much	Much	Too much	Total
	23.7	252	30.2	12.7	5.2	100
**General physical health index**	6.4	24.9	38.2	25.9	4.6	100

**Table 3.** Percentage distribution of scores obtained by respondents by mental health domain

	**Always**	**Many times**	**Sometimes**	**Rarely**	**Never**	**Total**
**Energy and vitality**	7.9	27.3	31.6	21.5	7.2	100
**Emotional health**	7.5	23.6	31.6	27.4	9.9	100
	Very high	High	Moderate	Low	Very low	Total
**Role limitation due to emotional problems**	15.4	22.4	19.7	19.2	23.3	100
**Mental health index**	12.9	23.4	29.1	23.8	10.8	100

**Social health domain: **Two questions were developed to measure the social functioning index of health in the elderly: "To what extent has your physical and mental condition interfered with your normal social relationships with family, friends, neighbors, or others during the past four weeks?" and "To what extent have physical and emotional problems interfered with your social activities during the past four weeks?" Then, the sum of the results of the responses to these two questions was divided into 5 categories. The results are shown in the table below.

**Quality of life index for the elderly: **The QOL of the elderly was measured using 4 components (well-being, life satisfaction, presence of a sense of purpose in life, and fulfillment of needs) and 14 subcomponents. The results in the table below illustrate that about 30% of the elderly studied have a high QOL. The QOL of more than one-third of the elderly was moderate, and about 34% of them had low QOL.

**Table 4.** Frequency and percentage distribution of scores obtained by respondents by social health domain

**Percentage**	**Frequency**	**Social functioning score**
2.6	10	<20
14.4	56	20-40
31.3	122	40-60
36.7	143	60-80
15.1	59	>80
100	390	Total

**Table 5:** Percentage distribution of scores obtained by respondents by quality of life indicators

	**Very low**	**Low**	**Moderate**	**High**	**Very high**	**Total**
**Well-being**	11.1	20.7	38.1	19.2	11.0	100
**Life satisfaction**	12.3	29.1	40.0	14.4	4.3	100
**presence of a sense of purpose in life**	8.7	15.7	27.6	23.6	24.4	100
**fulfillment of** ** needs**	10.9	25.9	38.7	19.7	4.8	100
**Quality of life index**	10.7	22.9	36.1	19.2	11.1	100

**Testing the hypotheses**

**The first hypothesis:** There is a relationship between the physical, mental and social health of the elderly and the QOL. Pearson correlation method was used to answer this hypothesis. Table 6 represents the correlation coefficient between the physical, mental and social health of the elderly and the QOL. The results showed that there was a significant relationship between physical health (0.176), mental health (0.16), and social health (0.178) and QOL at the 0.01 level.

**Hypothesis 2: **The rate of healthy aging and its components vary by gender. Analysis of variance (ANOVA) was used to examine the difference between health and QOL indicators and to determine if there was a statistically significant difference between men and women. The results are demonstrated in the table below. According to the results of the above table, there is a significant difference between elderly men and women in terms of QOL, physical health, mental health and social health, so elderly men have higher have QOL, physical health, and social functioning than elderly women, but elderly women have higher mental health compared to elderly men. 

**Table 6:** Correlation coefficient between physical, mental and social health of the elderly and quality of life

**Variables**	**Physical health**	**Mental health**	**Social health**	**Quality of life**
**Physical health**	**-**			
**Mental health**	0.678**	-		
**Social health**	0.47**	0.57**	-	
**Quality of life**	0.176**	0.16**	0.178**	**-**

**There was a statistically significant relationship between the physical, mental and social health of the elderly and quality of life (P˂0.001)

**Table 7:** Independent t-test to evaluate the difference in research variables among the elderly by gender

	**t-statistic**	**Degrees of freedom**	**significance level**	**Mean difference**	**Minimum**	**Maximum**
**Physical health**	-4.84	388	0.001	8.65	-12.16	-5.14
**Mental health**	2.67	388	0.001	4.27	1.12	7.41
**Social health**	-5.02	388	0.001	10.07	-14.01	-6.13
**Quality of life**	-4.79	388	0.001	5.64	-7.96	-3.33

## Discussion

Successful aging, that is, the degree of adaptation to old age, means that older people can maintain their physical and emotional health and have good family and social relationships even in old age. In the process of aging, older people experience mental perceptions and emotional reactions (aging perceptions) in the face of physical, mental, and social aging. The positive and negative perceptions of aging affect the behavioral tendencies of older people during the aging process (15). 

Positive aging perception refers to the positive effects and experiences of aging, whereas negative aging perception refers to the negative experiences of the mental, physical, and social aspects of aging (16). For older people, the perception of aging is an important factor that affects their physical and mental health. A positive perception of aging has a positive impact on their physical and mental health, whereas a negative perception of aging has a negative impact on their physical and mental health (17). 

QOL is a very abstract, multi-faceted concept influenced by time and place, and while it has objective dimensions and depends on external, mental, and internal conditions, it also depends on the individual's perception of his or her life situation. In a national survey of the QOL of 999 older people in the United Kingdom and Scotland, researchers found that older people who rated their QOL negatively attributed their reduced social contacts to the death of friends and family, resulting in a lower QOL (18). The current study investigated the physical, mental and social health and QOL of elderly people in Mazandaran and examined the relationship between health indicators of elderly people and their QOL, as well as the difference between these indicators among the elderly men and women studied.

In the ongoing study, 76.4% of the elderly had average and above average general health status, which was higher than the international average, consistent with the findings of Hosseini et al. (19) and Asadi Brojen et al. (20). Regarding physical performance, 59.3% of the study participants were at an intermediate and higher level and their performance was satisfactory, which is in agreement with the results of Mojahed et al. in Sistan and Baluchestan (21) and Hosseini et al. in Babol (19). In bodily pain that limits the physical and mental functioning and social role of the elderly, about 20% of people suffer from severe and more severe pain, mostly due to musculoskeletal involvement, and the most important solution is early diagnosis, physiotherapy and regular age-appropriate exercise programs. In a study conducted by Mirzamani et al., the severity of pain resulted in impairment of daily activities due to pain and increased dependence on spouse and family with a decrease in general activities (22). 

Moreover, in the present study, there was a direct and significant relationship between mental health and QOL, which means that loneliness and social isolation in the elderly are associated with a deterioration of physical and mental health, leading to a lower QOL. This finding has been demonstrated in several studies (23, 24). In the current study, about 37% of the elderly had impaired emotional health, especially in the areas of loneliness, depression, and Alzheimer's symptoms, and about 28.7% of the elderly rarely felt vital and alive. In a study conducted by Saeidimehr et al. in Ahvaz in 2015, it was shown that depression leads to a decrease in QOL and that building reassuring relationships and engaging the elderly in constructive activities boosts their self-confidence and increases their QOL (25, 26). Sheikholeslami et al. in Guilan pointed out that loneliness affects the overall health of the elderly and recommended that health care team members should be aware of the complications and consequences of loneliness and its impact on health (27). 

In the ongoing study, a direct and significant relationship was found between social relationships and QOL. In the area of social functioning and health of the elderly, impaired relationships with others (including family, friends, and neighbors) and social activities, 9.7% and 24.4% of the elderly had impaired relationships with others and impaired social activities, respectively, mainly due to physical, emotional, and mental problems and lack of social and family support. In a study conducted by Farzaneh in 2013, it was shown that there was a significant relationship between social support (including friends, family, and others) and social health, with family support having the largest and strongest influence (28). Melchiorre et al. (2013) suggested that older people living in large homes with spouses or others were more likely to experience high levels of social support (29). The study by Hekmatipour et al. revealed that 59% of the factors affecting the QOL of older people were related to social support, which mainly affects the psychological dimension of QOL and has no effect on the physical and social dimensions (30).

Anything that reduces a person's self-esteem and social value can contribute to the development of mental disorders in the elderly. Forced retirement, along with a deep respect for work, can cause negative feelings toward oneself and others, and these feelings can cause physical and psychological problems. Emotional difficulties can have many other social causes, such as loss of income, feeling that one is no longer the master of one's own destiny, change of residence, or health problems (31).

The main result of these changes seems to be loneliness, as all of these elements reduce social contact. Loss of self-esteem actually leads to the feeling that the person is no longer communicating with others as a human being. Loneliness can be defined as a personal state or feeling that occurs when a person estimates that his or her level of social relationships is insufficient or unsatisfactory. Loneliness may be associated with a kind of shame and embarrassment that prevents a person from taking the initiative for social contact after a change of environment (32). Numerous studies (Jafari et al. in Mazandaran and Ricardo in Brazil) point out the negative effects of loneliness, stress, and depression on the QOL of older people (23).

Gender is one of the determinants of QOL. In the present study, there was a significant difference between older men and women in QOL, physical health, mental health, and social functioning, such that older men had higher QOL, physical health, and social functioning than older women, but older women had higher mental health compared to older men. In most other studies, QOL was lower in older women than in older men (33, 34), while the results of the study by Khaje et al. showed that QOL had no significant association with gender (35). 

As a general conclusion, the elderly have always been neglected as an important population group in society, while the issues and problems related to aging are becoming more important as their population grows. As men and women grow older, they are rejected and ignored by their families, workplaces, and social groups. On the other hand, older people are vulnerable to exclusion from society because of their individual characteristics.The ongoing study has indicated that the health indicators of the elderly residents in Mazandaran are at a moderate to low level. Theoretical and experimental research have shown that factors such as timely marriage and strong family relationships (36), education level (especially health literacy) (37), social participation (29), socioeconomic status (38), family support (38), and loneliness (39) are the most important factors affecting the health status of the elderly in the mental, social and physical domains. 

It is hoped that the results of the current study will provide a basis and direction for conducting further studies and an appropriate context for interventions to improve the QOL of this vulnerable group. In the present study, the description of the QOL of the elderly is emphasized. It is suggested that further studies should be conducted to evaluate the QOL of sick elderly people, and that future researchers should conduct a similar study in other cities and provinces in the country so that more confidence can be gained in the use of these results by comparing the results of different researches in this area. In addition, future studies should also investigate other factors influencing the improvement of health levels of the elderly. Since the Iranian population has been very young in recent years, most planning has focused on youth issues, but the growing trend in the elderly requires further study and planning. As a rule, there are always limitations in research dealing with the study and understanding of human activities, and the problem of collecting information from individuals always arises. The present study is based on the statistical population of elderly people in Mazandaran, so its generalization to other societies should be done with greater caution and care.
